# Does interferential current provide additional benefit to orthopedic rehabilitation for the patients with proximal humeral fractures? A randomized controlled study

**DOI:** 10.1186/s12891-024-07232-4

**Published:** 2024-02-07

**Authors:** Emine Duran, Berrin Durmaz, Funda Çalış Atamaz, Mehmet Resul Kadı, Levent Küçük

**Affiliations:** 1 Department of Physical Medicine and Rehabilitation, Medical School of Ege University, Izmir, Turkey; 2 Department of Orthopaedic Surgery, Medical School of Ege University, Izmir, Turkey

**Keywords:** Rehabilitation, Humeral fractures, Interferential current therapy, Shoulder pain

## Abstract

**Background:**

Approximately 80% of all proximal humeral fractures (PHFs) are non-displaced or minimally displaced fractures, which can be treated with conservative treatment. This study investigated the effect of interferential current (IFC) added to orthopedic rehabilitation on shoulder function, pain, and disability in patients with PHF.

**Methods:**

This study was a prospective, double-blind, randomized, placebo-controlled conducted in physical medicine and rehabilitation outpatient clinic. Thirty-five patients were randomly separated into the IFC group (*n* = 18) and the sham group (*n* = 17). The orthopedic rehabilitation program was applied to all patients by the same physiotherapist three times a week for four weeks. Patients in the IFC group received the intervention for 20 minutes 3 times a week before the exercise. The same pads were performed for the sham group, but no electrical stimulation was applied. Constant-Murley score (CMS) for shoulder function, visual analog scale (VAS) activity pain, disabilities of the arm, shoulder, and hand (DASH) score, and paracetamol intake were recorded post-treatment, at 6 weeks and 18 weeks post-treatment.

**Results:**

The demographic and fracture characteristics were not different between the groups. Significant differences were observed in the IFC and sham group in intragroup comparisons of total CMS, VAS activity pain, DASH score, and paracetamol intake over time (*p* < 0.001). Significant improvement over time was valid for all pairwise comparisons in both groups. However, no significant differences were detected between the IFC and sham group.

**Conclusion:**

IFC added to orthopedic rehabilitation could not appear to be an electrotherapy modality that could potentially benefit shoulder function and disability in patients with PHF.

## Introduction

Proximal humerus fractures (PHFs) frequently occur in the elderly and osteoporotic population, but these fractures are also common in individuals under 60 years of age [1]. Although appropriate treatment depends on the specific characteristics of the fracture and patient, conservative treatment for non-displaced or minimally displaced PHFs leads to good outcomes in 80 to 90% of patients. Also, functional results have been achieved with conservative treatment in selected cases with displaced fractures [2]. Conservative treatment of PHF usually involves a short period of immobilization followed by orthopedic rehabilitation [3]. However, the severe pain of some patients with fractures limits their participation in the exercise program, and shoulder muscle atrophy and frozen shoulder may occur in these patients due to immobilization. There are conflicting results about the use of physical therapy modalities in shoulder pain management [4]. Two randomized controlled studies showed that interferential current (IFC), an electrotherapy modality used commonly for the treatment of shoulder pain, was effective in patients with frozen shoulder. In the first of these studies, an increase in shoulder function and a decrease in pain scores were obtained in the exercise plus IFC group compared to the control group. The second study compared IFC or ultrasound therapy added to hot pack plus exercise and showed that IFC was more effective in increasing shoulder ROM. However, the absence of a group that received only standard treatment in both studies was an important limitation [5, 6]. In contrast to frozen shoulder, two randomized controlled trials indicated that IFC added to standard treatment in subacromial impingement syndrome was no different from sham or standard treatment [7, 8].

The basic principle of IFC is a medium-frequency electrical current amplitude-modulated in low frequency, generated by the superimposition of two medium-frequency currents slightly out of phase [9]. IFC therapy is believed to be effective for the pain-relieving through several mechanisms, including gate control and release of endogenous opiates [10]. Also, the placebo effect is a condition that can not be neglected [11]. Although IFC has been investigated in many painful shoulder disorders, there is no reported study on the effectiveness of IFC therapy in patients with PHF.

This study aimed to investigate the effectiveness of IFC added to exercise on shoulder function, pain, and disability compared with placebo in patients with conservatively treated PHF.

## Methods

### Trial design

This study was a randomized controlled trial in which both patients and assessor were blinded, following all the Consolidated Standards of Reporting Trials (CONSORT) recommendations. Participants were recruited from physical medicine and rehabilitation outpatient clinic. The study protocol was approved by Ege University Ethics Committee (decision number 14–1/13) and registered on ClinicalTrials.gov (NCT04553497).

### Participants

The initial recruitment consisted of 53 patients aged 40–80 years with non-displaced PHF and not operated displaced PHF. Fracture type was staged according to Neer classification [12]. Inclusion criteria were as follows: age ≥ 40 years, PHFs did not require surgery by the orthopedic surgeon, and admission to our outpatient clinic within the first two weeks after the fracture. Patients were excluded from the study if they met any of the following exclusion criteria: any surgery history for shoulder pathologies; previous electrotherapy experience before the fracture (to ensure blinding of therapy); any contraindication for IFC (pacemaker, malignancy, pregnancy, active thrombosis or thrombophlebitis, untreated hemorrhagic conditions, active infected tissues); rheumatic diseases such as rheumatoid arthritis and ankylosing spondylitis; shoulder subluxation; having other fractures in addition to the PHF; known or suspected joint infection or a specific condition such as peripheral or central nervous system lesions; neoplasm; diabetes mellitus or osteonecrosis; any mental disorder that may make it difficult to adapt to exercise. All inclusion and exclusion criteria were fulfilled by a physiatrist experienced in orthopedic rehabilitation (ED). All patients were briefed about the study, and written consent was obtained.

### Rehabilitation program

All patients were included in the study within the first week of PHF. The orthopedic rehabilitation program was applied to all patients three times a week for  four weeks under the guidance of the same physiotherapist. The patients also received a complete set of premade exercise cards, which showed all exercises to ensure that the training program was learned correctly. The fractured shoulder was immobilized with a Velpeau bandage or sling for 3 or 4 weeks, except for exercise.

The first phase of the rehabilitation (0–3 weeks) involved the elbow, wrist, and hand active range of motion (ROM) and pendulum (clockwise and counterclockwise) exercises in the 0–2 weeks of the non-displaced fracture. For displaced fractures, elbow, wrist, and hand active range of motion was started immediately, but pendulum exercises were initiated two weeks later. The patients were instructed to continue exercises 3–5 times per day for 30 minutes each session. After two weeks, active assistive ROM and isometric exercises were performed in the supine position. During the second phase (3–6 weeks), active forward elevation in supine was carried out and then progressed to sitting and standing position. At the end of the sixth week, a home exercise program was given by the physiotherapist, including resistance exercises for internal and external rotation, flexion, extension, and abduction using an elastic band (Thera-Band). Flexibility and stretching exercises were also given to increase ROM progressively in all directions [2, 13, 14]. The patients were instructed to perform their exercises regularly at each visit.

### Interventions

Patients were evaluated within the first week of PHF and divided into two groups to receive either IFC or sham using a simple randomization method. IFC was applied three times a week for 20 minutes before each exercise session by another physiotherapist. The IFC therapy was applied using a combined electrotherapy device SONOPULS 692® (brand: Enraf-Nonius). The medium frequency of the IFC was 4000 Hz and 4100 Hz to produce the amplitude-modulated frequency at 100 Hz. Two rubber electrodes (8 × 6 cm) were fitted on the fractured shoulder. One of the electrodes was placed on the lateral part of the deltoid muscle; the other one was placed on the trapezius muscle near the shoulder. The current intensity was set to achieve a “strong but comfortable tingling” without visible muscle contraction [15, 16]. The sham therapy consisted of placing the same pads for the same duration, but no electrical stimulation was applied to the probes.

### Outcome measures

The primary outcome was global shoulder function which was measured by the Constant-Murley score (CMS). It assesses four shoulder functions: pain (15 points), activities of daily living (sleeping, work, leisure) (20 points), range of motion (40 points), and muscle strength (25 points). The total score ranges from 0 to 100, with the higher score indicating better shoulder function [17].

Activity pain was measured with the visual analog scale (VAS) (0-10; 0 = no pain and 10 = the worst pain). In addition, in order to evaluate shoulder function and disability, the Disability of the Arm, Shoulder, and Hand questionnaire (DASH) was used [18]. The DASH questionnaire measures the physical function and symptoms of patients with musculoskeletal disorders in the upper limb. It consists of 30 items: 6 items about symptoms and 24 items about function. Patients answer the questions using a 5-point Likert system, and the cumulative score is scaled from 0 to 100, with higher scores indicating more disability. The patients were allowed to use only paracetamol during the study, and the paracetamol intake was recorded as gram/week. All outcome measures were evaluated immediately post-treatment, at 6 weeks, and 18 weeks post-treatment by the physiatrist (ED) who did not know which group the patients belonged to. Only VAS resting pain was evaluated at the enrollment because the fractured side was immobile when the patients were included in the study.

### Sample size calculation

A 15-points difference in the Constant-Murley score was considered significant in the comparison between the groups after intervention, assuming that a standard deviation of 17 [19, 20]. Taking into account a desired power of 80%, an alpha value of 0.05, and a high effect size (d = 0.88) were presumed. Assuming a dropout rate of 5%, at least 17 patients were required per study group (G*Power, version 3.1.9.2, Heinrich Heine University, Düsseldorf, Germany).

### Randomization, allocation concealment, implementation, and blinding

The patients were recruited within the first week of PHF and divided into two groups using a simple randomization method managed by an impartial observer. Flipping a coin was used for simple randomization (i.e., heads - sham, tails - treatment). The patient’s group was reported to the physiotherapist who would apply IFC in a closed envelope. Each participant was unaware of the allocation of the group and received sham or active IFC therapy. In addition, the outcome assessor were also blind to the treatment groups.

### Statistical methods

Statistical analysis was performed using SPSS version 20.0 (IBM, Armonk, NY, USA). An intention-to-treat analysis was employed for all analysis. The variables were investigated using visual (histogram, probability plots) and analytic methods (Kolmogorov-Smirnov, skewness and curtosis) to determine whether they were normally distributed or not. Continuous data were described as median (inter-quartile range, IQR) or mean (standar deviation, SD) and categorical variables as percentages. Chi-square or Fisher’s exact test was used to compare categorical variables and Mann-Whitney U test/Student’s T-test was used to compare continuous variables. All outcome values were presented in mean and standard deviation. To analyze the between-groups and within-groups differences on outcomes, repeated measures analysis of variance (ANOVA) was used for the parametric data using the interaction terms “group vs. time” with Bonferroni post-hoc test with adjusted *P* values. The results of the repeated measures ANOVA were analyzed by Mauchly’s sphericity test. If the parametric tests (factorial design for repeated measures analysis) did not provide the preconditions, the Greenhouse-Geisser correction was used for corrections to the degrees of freedom or Friedman’s Test. The Bonferroni correction was used for multiple comparisons. A *p* value of less than 0.05 was considered to show a statistically significant result.

## Results

### Study population and patient characteristics

**Fig. 1:**
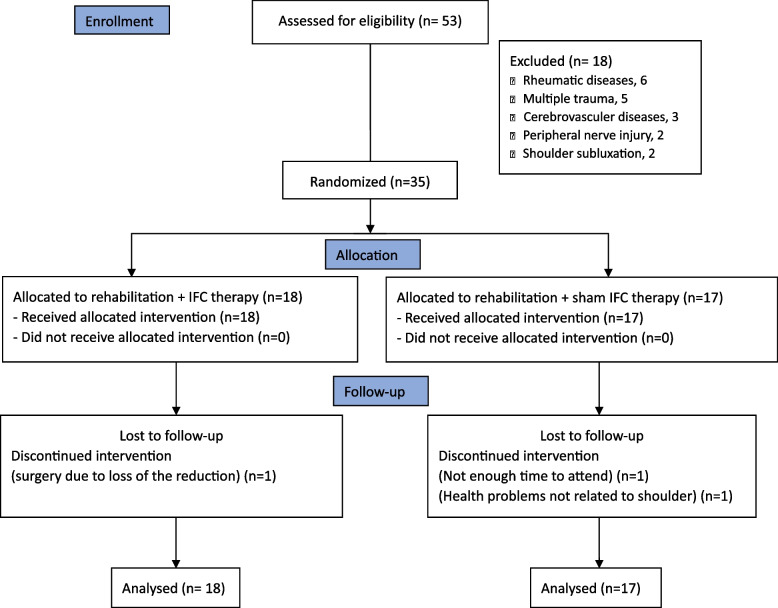
CONSORT diagram of flow of the participants in the study

A total of 53 individuals were recruited for the present study. Of these, 18 were excluded from the study due to exclusion criteria. Thirty-five patients were included in the study in the first week after fracture and divided into two groups: 18 patients in the IFC group (rehabilitation+IFC) and 17 patients in the sham group (rehabilitation+sham IFC). One patient in the IFC group and two patients in the sham group dropped out during the follow-up. Thirty-two patients completed the study. A CONSORT diagram of the study was presented in Fig. 1. None of the patients who completed the study reported any adverse effects. There were no differences between the groups in terms of age, gender, body mass index (BMI), fractured and affected side, Neer classification type, fracture anatomical segment, and VAS rest pain (Table 1).

**Table 1:** General characteristics of the participants and fractures

Variables^a^	IFC group (*n* = 18)	Sham group (*n* = 17)	p
Age, year (mean ± SD)	58.9 ± 10.7	62.0 ± 9.5	0.381
Gender			
Female	12 (66.7)	11 (64.7)	0.813
Male	6 (33.3)	6 (35.3)	
Education level			
Primary school or less	11 (61.1)	13 (76.4)	
High school	4 (22.2)	2 (11.8)	0.645
College	3 (16.7)	2 (11.8)	
BMI (mean ± SD)	28.0 ± 3.2	30.8 ± 5.6	0.103
Presence of comorbidity	9 (50)	12 (70.6)	0.214
Number of comorbidities			
One	5 (27.8)	4 (23.5)	0.273
Two or more	4 (22.2)	8 (47)	
History of osteoporosis	5 (27.8)	3 (17.6)	0.539
Regular exercise habit	2 (11.1)	2 (11.8)	0.998
Fractured side			
Right	15 (83.3)	15 (88.2)	0.173
Left	3 (16.7)	2 (11.8)	
Effected Side			
Dominant	9 (50.0)	11 (64.7)	0.942
Non- dominant	9 (50.0)	6 (35.3)	
Neer Classification			
Type 1	4 (22.2)	2 (11.8)	
Type 2	3 (16.7)	6 (35.3)	0.125
Type 3	11 (61.1)	9 (52.9)	
Anatomic segment			
Greater tuberosity	5 (27.8)	3 (17.6)	
Surgical neck	2 (11.1)	5 (29.4)	0.456
Greater tuberosity and surgical neck	11 (61.1)	9 (52.9)	
VAS resting pain (med, IQR)	8 (1.8)	8 (1)	0.891

*BMI* Body mass index, *IFC* Interferential current, *SD* standard deviation, *VAS* Visual analog scale

^a^n(%), if otherwise specified

### Outcome measures within and between the groups after intervention

**Fig. 2:**
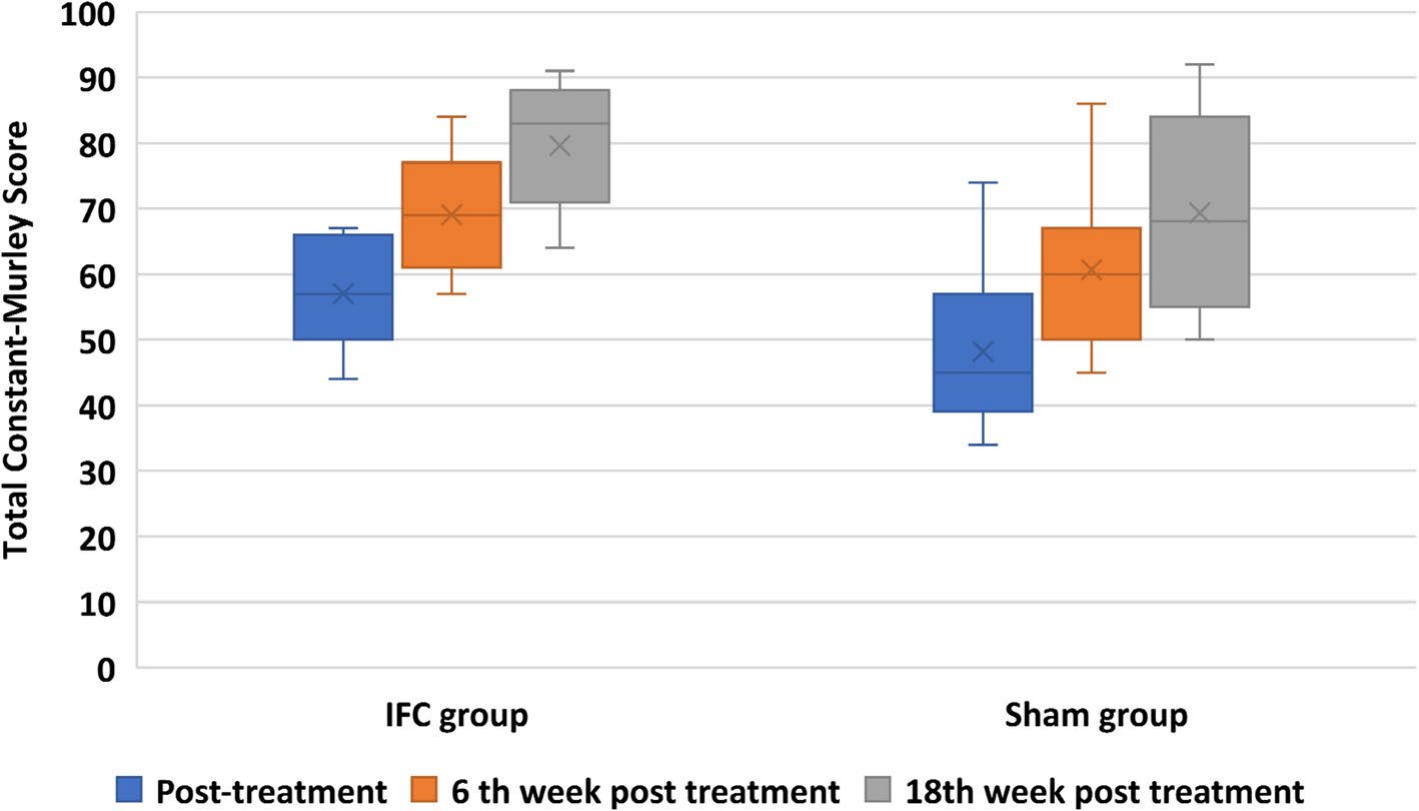
Total Constant-Murley Score of the fractured side within and between the groups

Significant differences were observed in the IFC and sham group in intragroup comparisons of total CMS and its subscores over time (*p* < 0.001). Significant improvement over time was valid for all pairwise comparisons (post treatment - 6th week, post treatment - 18th week, and 6th week -18th week) for both groups. However, no significant differences were detected among the groups (Table 2 and Fig. 2). Analysis of the VAS activity pain, DASH scores, and paracetamol intake were given in Table 3. VAS activity pain, DASH scores, and paracetamol intake decreased significantly over time in both groups (*p* < 0.001). Similarly, all pairwise comparisons were significant in the IFC group and the sham group and there were no distinction between the groups.

**Table 2:** Comparison of Constant-Murley Score and its subscores within and between the groups

Total CMS (mean, SD)	IFC group (*n* = 18)	Sham group (*n* = 17)	p^**^
Post-treatment	57 ± 7.7	48.2 ± 12	0.727
6th week post-treatment	69 ± 8.9	60.7 ± 12.1	
18th week post-treatment	79.6 ± 9.4	69.3 ± 14.2	
p^*^	**< 0.001**	**< 0.001**	
**CMS subscores**			
**Pain**			
Post-treatment	9.1 ± 3.2	7.9 ± 4.8	
6th week post-treatment	11.8 ± 2.5	9.7 ± 3	0.667
18th week post-treatment	13.8 ± 2.2	12 ± 3.2	
p^*^	**< 0.001**	**< 0.001**	
**Activity level**			
Post-treatment	13.4 ± 1.7	12.2 ± 3.7	
6th week post-treatment	16.7 ± 3.1	14.8 ± 3.1	0.775
18th week post-treatment	19.2 ± 1.7	17.5 ± 2.6	
p^*^	**< 0.001**	**< 0.001**	
**ROM**			
Post-treatment	27.5 ± 3.6	24 ± 4.3	
6th week post-treatment	30.8 ± 4.5	27.2 ± 4.3	0.09
18th week post-treatment	34.9 ± 5.1	29.1 ± 4.5	
p^*^	**< 0.001**	**< 0.001**	
**Strength**			
Post-treatment	6.4 ± 3	4.7 ± 4	
6th week post-treatment	9.8 ± 3.2	9 ± 5	0.425
18th week post-treatment	12.2 ± 4.2	11.4 ± 5.5	
p^*^	**< 0.001**	**< 0.001**	

*CMS* Constant-Murley Score, *IFC* Interferential current, *ROM* Range of motion, *SD* Standard derivation

p^*^ Intragroup changes over time

p^**^ Intergroup interaction over time

**Table 3:** Comparison of VAS activity pain, DASH scores, and paracetamol intake within and between the groups

VAS activity pain (mean, SD)	IFC group (*n* = 18)	Sham group (*n* = 17)	p^**^
Post-treatment	3.9 ± 1.5	4.7 ± 1.5	
6th week post-treatment	2 ± 1.7	3 ± 1.5	0.793
18th week post-treatment	0.7 ± 1.1	1.7 ± 1.4	
p^*^	**< 0.001**	**< 0.001**	
**DASH scores**			
Post-treatment	28 ± 11	38.4 ± 17.5	
6th week post-treatment	15.3 ± 9.2	23.3 ± 13	0.299
18th week post-treatment	7.7 ± 7.3	12.5 ± 11.3	
p^*^	**< 0.001**	**< 0.001**	
**Paracetamol intake (g/week)**			
Post-treatment	9.7 ± 7.6	11 ± 9.2	
6th week post-treatment	3.7 ± 4.5	6.7 ± 8.2	0.583
18th week post-treatment	1.2 ± 2.2	4.7 ± 6.7	
p^*^	**< 0.001**	**< 0.001**	

*DASH* The Disability of the Arm Shoulder and Hand, *IFC* Interferential current, *SD* Standard derivation, *VAS* Visual analog scale

p^*^ Intragroup changes over time

p^**^ Intergroup interaction over time

## Discussion

This randomized, placebo-controlled, and prospective study demonstrated that IFC therapy added to rehabilitation program did not significantly improve shoulder function and disability compared with sham therapy in patients with PHF. However, the patients in both groups achieved good shoulder function at the end of the follow-up period. This result reveals the importance of early mobilization and orthopedic rehabilitation programme.

Conservative treatment provides satisfactory results in non-displaced and selected displaced PHF [2]. Previous studies comparing the initiation of physiotherapy within one week after fracture versus delayed physiotherapy after three weeks of immobilization reported that the early group had significantly better shoulder function and health-related quality of life scores [14, 19, 21]. Additionally, conservative treatment with an effective rehabilitation program has been reported to be successful even in displaced PHF of elderly patients with low functional capacity [22, 23]. Although surgical treatment is superior to conservative treatment in displaced PHFs, surgical treatment should be considered in selected patients due to the high incidence of postoperative complications [2, 24]. In our study, although more than half of the patients had 3-part fractures, the good shoulder functional results we obtained at the end of follow-up revealed the importance of early mobilization and effective rehabilitation.

The information about IFC therapy is limited in patients with PHF. In the literature, we have found only one abstract of a study evaluating the effect of IFC therapy on PHFs. This study stated that IFC did not provide additional benefits to shoulder function in addition to exercise. However, it turned out that this study was not published due to the discovery of problems with randomization [15]. In studies published on other shoulder pathologies, IFC combined with shoulder exercises was established to be more effective than the no treatment group in patients with frozen shoulder [5]. Similarly, another randomized controlled study showed that IFC added to standard treatment was more effective in increasing ROM than ultrasound therapy in patients with frozen shoulders [6]. However, the absence of a group receiving only standard treatment in both studies neglects the placebo effect of IFC. In subacromial impingement syndrome population, it has been shown that IFC added to exercise did not add any significant value when compared with standard treatment alone or with placebo [7, 8]. In the current literature, the  insufficiency of studies evaluating the effectiveness of IFC in PHFs and conflicting results in other shoulder pathologies make it difficult to comment on the effectiveness of IFC. In our study, similar improvement in shoulder functions was achieved in both the IFC group and the sham group. This result may be attributed to both the exercise program started in the early period and the placebo effect of IFC.

Although IFC therapy has been used for the last several decades, its physiological effects have not been sufficiently established to explain the analgesic effect. Some theories, such as gate control theory, descending pain suppression pathway, physiological blockade and placebo effect, are proposed to explain the analgesic effect [11]. In a recent systematic review evaluating the effect of IFC on musculoskeletal pain, IFC alone was shown to be statistically but not clinically effective in reducing pain compared to placebo [9]. Two recently published randomized controlled trials compared IFC or sham therapy added to exercise plus hot pack with standard treatment in patients with subacromial impingement syndrome; as a result of these studies, they reported that there was no difference between the groups in terms of pain and shoulder disability scores [25, 26]. In our research, IFC added to the exercise did not contribute additional effect for pain relief. However, the absence of only exercise group in our study neglects the placebo effect on a subjective parameter such as pain.

In our study, the similarity in shoulder function scores was also reflected in the DASH score, which indicates shoulder disability. Although DASH scores were higher in the sham group at all three visits, the improvement over time was not different in both groups. It was not surprising that disability scores were similar in PHF patients with similar shoulder function and pain scores. It has also been reported that the DASH score may be affected by upper extremity compensatory mechanisms [27].

Some strengths and limitations of our study need to be addressed. Firstly, we could not present the baseline data for CMS and DASH scores because the fractured shoulder was immobilized with a Velpeau bandage or sling before the intervention. This may have biased our results. Additionally, the lack of a third group that included only the exercise intervention to evaluate the placebo effect of IFC was also an important limitation. The presence of an only exercise group could reveal whether there was a placebo effect. Another limitation was the small sample size of the study. Despite all these limitations, major strength of our study was that it was a double-blind, randomized, placebo-controlled study involving patients with proximal humerus fracture and had follow-up data for more than four months.

## Conclusion

Conservatively treated patients with displaced and non-displaced PHF did not achieve better shoulder function, pain, and disability scores with the addition of IFC to orthopedic rehabilitation compared to sham therapy. Although IFC does not appear to be an electrotherapy modality that could potentially benefit shoulder function in patients with PHF, further randomized controlled studies with long-term follow-up and large sample size are needed to evaluate the effectiveness of IFC in patients with PHF.

## Data Availability

The datasets generated during and/or analysed during the current study are not publicly available but are available from the corresponding author on reasonable request.

## Abbreviations

CMS: Constant-Murley score
CONSORT: Consolidated Standards of Reporting Trials
DASH: Disabilities of the arm, shoulder, and hand
IFC: Interferential current
PHFs: Proximal humeral fractures
VAS: Visual analog scale
